# Cell properties assessment using optimized dielectrophoresis-based cell stretching and lumped mechanical modeling

**DOI:** 10.1038/s41598-020-78411-1

**Published:** 2021-01-27

**Authors:** Imman I. Hosseini, Mahdi Moghimi Zand, Amir Ali Ebadi, Morteza Fathipour

**Affiliations:** 1 Small Medical Devices, BioMEMS & LoC Lab, Department of Mechanical Engineering, College of Engineering, University of Tehran, Postal Code 14399-55961 Tehran, Iran; 2 MEMS & NEMS Lab, School of Electrical and Computer Engineering, Faculty of Engineering, University of Tehran, Tehran, Iran

**Keywords:** Engineering, Mathematics and computing, Nanoscience and technology, Physics

## Abstract

Cells mechanical property assessment has been a promising label-free method for cell differentiation. Several methods have been proposed for single-cell mechanical properties analysis. Dielectrophoresis (DEP) is one method used for single-cell mechanical property assessment, cell separation, and sorting. DEP method has overcome weaknesses of other techniques, including compatibility with microfluidics, high throughput assessment, and high accuracy. However, due to the lack of a general and explicit model for this method, it has not been known as an ideal cell mechanical property evaluation method. Here we present an explicit model using the most general electromagnetic equation (Maxwell Stress Tensor) for single-cell mechanical evaluation based on the DEP method. For proof of concept, we used the proposed model for differentiation between three different types of cells, namely erythrocytes, peripheral blood mononuclear cells (PBMC), and an epithelial breast cancer cells line (T-47D). The results show that, by a lumped parameter that depends on cells' mechanical and electrical properties, the proposed model can successfully distinguish between the mentioned cell types that can be in a single blood sample. The proposed model would open up the chance to use a mechanical assessment method for cell searching in parallel with other methods.

## Introduction

Subcellular components such as the cytoskeleton, lipid bilayer membrane, cytoplasm, focal adhesion proteins, and extracellular matrix (ECM) are integral components of the cell structure and mechanics in health and disease cells^1^. Pathogens, disease conditions, and therapeutic interventions may influence the subcellular components’ mechanobiological properties and structural arrangement and organization^2^. For example, Malaria inducing Plasmodium falciparum increases the cytoplasm's stiffness noticeably by producing inert crystals of hemozoin^3^. Also, the cell's biomechanical properties are impacted by cancer and chemotherapy, for instance, affecting the polymerization of microtubule filaments and hence markedly influencing the cytoskeletal stiffness^4^. Furthermore, during epithelial-mesenchymal transitions (EMTs), cytoskeleton, and, consequently, overall cell biomechanics experience a remarkable change^5^. It is worth to be mentioned that EMT has been considered as a decisive factor for the CellSearch system^6^, which is the only FDA-approved system for the detection of the circulating tumor cells (CTCs)^7^. Therefore, assessing single-cell mechanical property can be a label-free method for pathology studies, including cancer detection.

A useful method for measuring cell biomechanical properties must be ideally high-throughput. In other words, it should be able to analyze a significant number of cells simultaneously, given the typical heterogeneity of cells in a tumor tissue. It must be also applied to various cells (e.g. adherent and nonadherent, nucleated and anucleated, stiff and soft, large, and small). Several methods for single-cell and cell population measurements have been proposed, including Atomic Force Microscopic (AFM)^8–10^, micropipette aspiration^11^, hydrodynamics methods^12^, and optical^13^ and magnetic tweezers^14^.

Each of the methods brings a series of advantages and disadvantages^9^. For example, the AFM method is only applicable to adherent cells, while the micropipette aspiration method is only applicable to the nonadherent cells. Furthermore, both AFM and micropipette aspiration suffer from low throughput. Although some efforts have been underway to overcome the deficiencies in these methods^15^, there are still other drawbacks, such as positioning cells at a predefined area or cost-effectiveness. On the other hand, the high throughput methods such as the hydrodynamics-based method are not appropriate for lab-on-chip (LOC) platforms due to their dependency on high frame rate cameras. Thus, we can conclude that further research in this field is required to propose an ideal lab-on-a-Chip method for high throughput cell mechanical properties assessment at a single-level.

Detection by electrical means have been successfully employed in cell-biology^16,17^. Different cells show different responses to an electrical field due to the differences in their dielectric properties. Among many electrokinetic detection methods, dielectrophoresis (DEP) has been widely used for cell separation and Molecular analysis^18–21^. DEP has also been used as a means of cell deformation^22–29^. It appears that this method can meet all the requirements stated above for an ideal method. However, there is no integrated model to investigate different cell deformation parameters induced by the DEP method. Although some efforts have been conducted to evaluate the effect of the parameters on cell deformation^8,30^, expensive simulation is required. We have widely studied the fundamentals of DEP-based MEMS before^31–35^. What is new in this research is an explicit equation based on the most general electromechanical model (Maxwell Stress Tensor) to differentiate different cell types using the DEP method. Here we show that different cell types can be distinguished by an exclusive lumped parameter that depends on cells' intrinsic properties. We examined three cell types for proof of concept, and the proposed lumped parameter is used to differentiate the cells sample. Furthermore, regarding the inconsistency in previous studies for the configuration of electrodes^22–27^, we studied different designs, and the optimum electrode is proposed based on optimization algorithms.

### Operational principles of the proposed microfluidic chip

**Figure 1:**
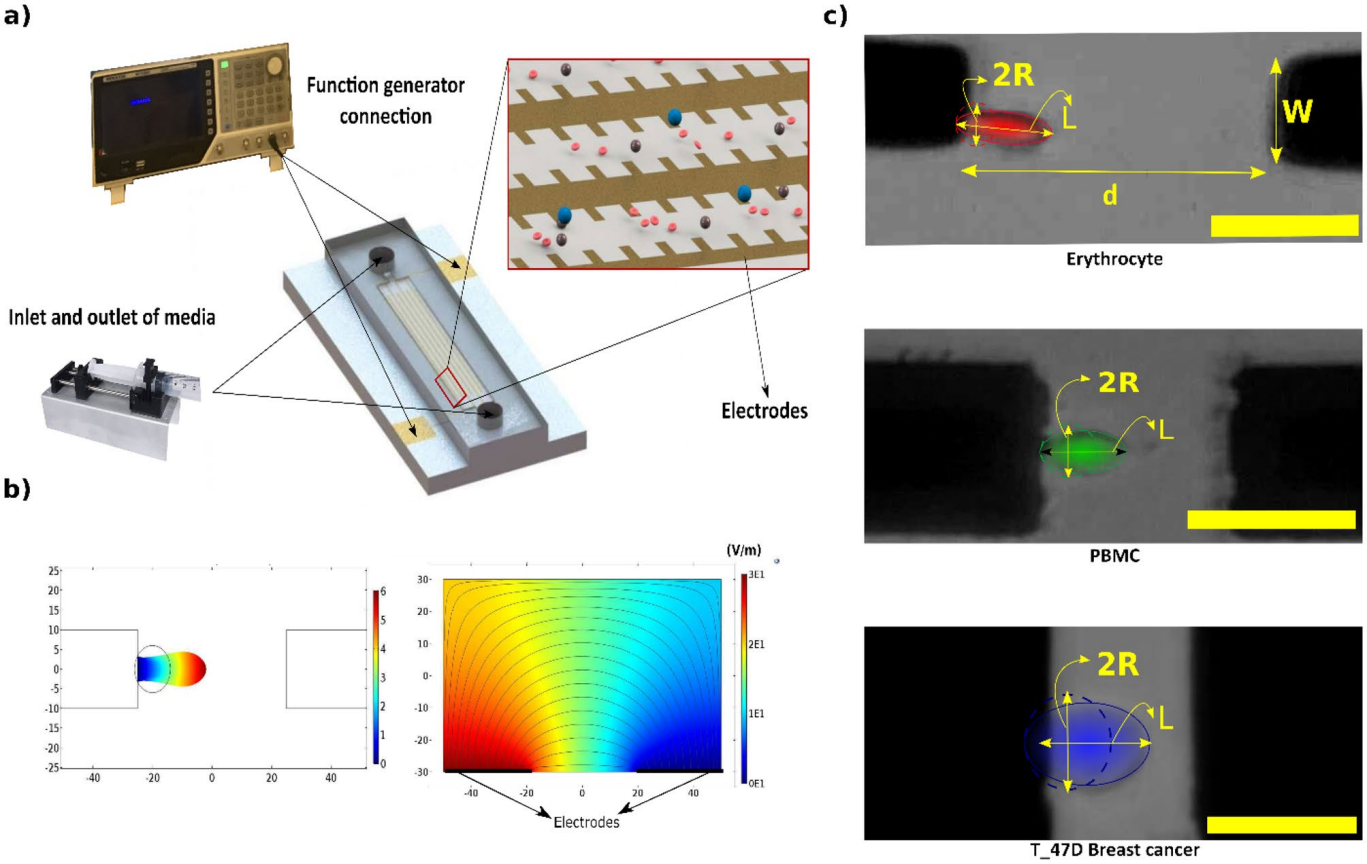
Operational principles of our proposed microfluidic chip (**a**) Schematic image of the microfluidic device and experimental setup. (**b**) Simulation results in single-cell deformation and electric field and electric field gradient in the channel's height. (**c**) Experimental pictures of elongated Erythrocyte, PBMC, and T-47D cells. The scale bar is 30 µm.

A dielectrophoresis-based micro-electromechanical chip is used for the evaluation of the mechanical properties of the cells. A schematic for the chip is given in Fig. 1a. The system is composed of two parts (1) a substrate with photolithographically patterned Ti/Au electrodes and (2) a microfluidic channel fabricated by soft lithography. A fluid containing suspended cells is introduced into the microfluidic channel, and excess fluid is discharged from the microfluidic channel into a waste chamber. A Differential potential can be applied to the electrodes using a Function Generator (F.G.). Once the channel is filled with dispersed cells, fluid flow is stopped.

Then, the F.G. is turned on, and cell elongation is captured using an inverted microscope and analyzed with an open-source image processing software (Tracker 5.1.5). The time-dependent cell elongation is evaluated for the investigation of the cells' mechanical properties. The applied voltage to the electrodes at the bottom of the channel induces a gradient of the electrical field in the z-direction (height of the channel) (Fig. 1b). The electric field attracts cells to the electrode. As shown in Fig. 1b, our simulation results are in line with our experimental results. The suspended cells, as well as fluid, are polarized by the applied electrical field. Due to the distinct dielectric properties of the cells and the medium, the cells may experience either stronger or weaker polarization depending on their relative dielectric properties in compare of the medium. Under such circumstances, cells undergo a non-equilibrium condition. Cells try to find an equilibrium state in the medium. In a uniform electric field, the cells' dipole forces reach an equilibrium state with internal mechanical forces, which results in the deformation of cells (Fig. 1c). The deformation depends on cells' intrinsic properties, such as geometry, permittivity, conductivity, and mechanical properties, as well as experimental conditions, including the applied voltage and geometry of electrodes.

The method allows employing a minute amount of sample for the effective detection of various kinds of cells. Most well-known previous methods only assess one of the intrinsic properties of cells, e.g. mechanical^8^, electrical^30^, or chemical^29^. As a result, they allow the detection of only a limited range of cell properties. For example, it has been shown that electrical properties can be used for distinguishing CTCs from leukocytes, but this method cannot be employed for the detection of cancerous cell types (e.g. mesenchymal or epithelial) since both cells show a similar electrical response. The proposed DEP method allows for the simultaneous analysis of both mechanical and electrical properties of the cells. Therefore, it can detect a wide range of cell properties. As mentioned earlier, this is the main superiority of this method over its counterparts.

## Results and discussion

### Experimental setup validation

**Figure 2:**
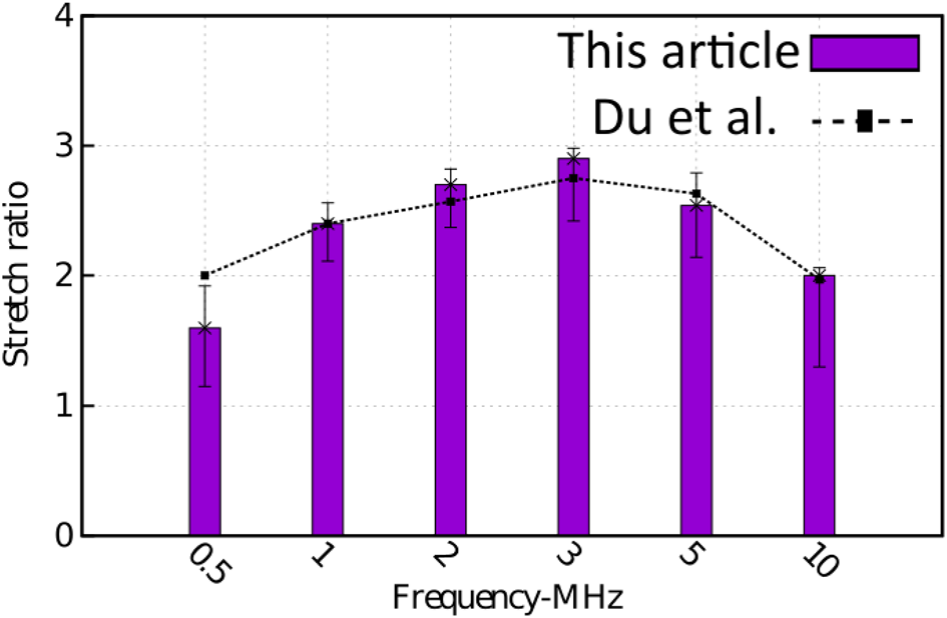
Comparison of our results and the results presented by Du et al.^24^ for the stretch ratio of erythrocytes for different frequency.

To validate our results, we compared our experiments and the results in Du et al.^24^. The results are presented in Fig. 2. In this figure, the strain of erythrocytes is depicted for different frequencies. As this figure shows, there is only a small difference between our results and the results presented in the previous study in Du et al.^24^.

### Cell viability

**Figure 3:**
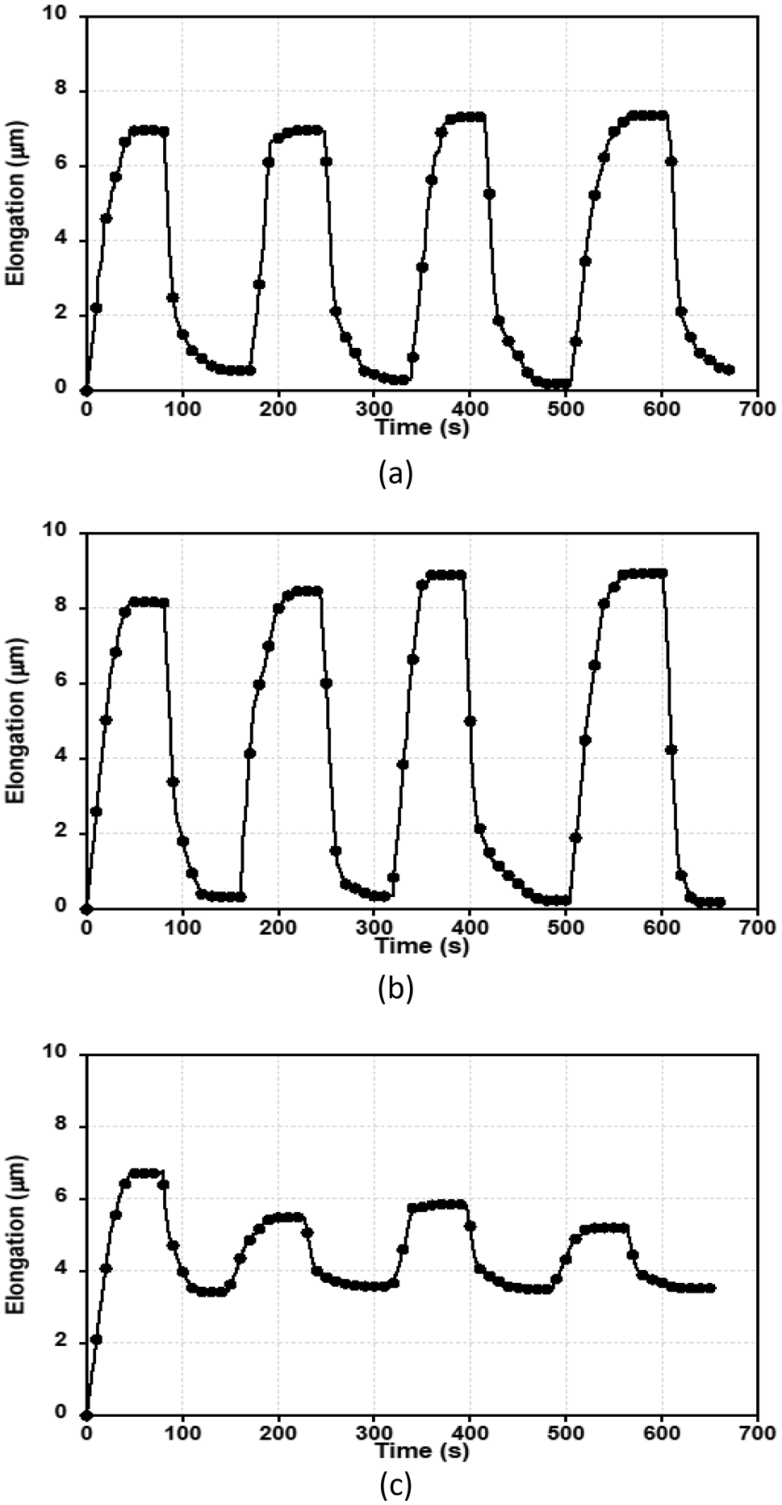
Experimental studies on cell viability for electrode voltages: (**a**) 8 V (**b**) 12 V (**c**) 16 V. In these figures, the elongation of cells is drawn versus time. At voltage 8 and 12 V, the cells have reversible and repeatable elongation. However, the cells at voltage 16 V, do not reverse to its original shape after turning off the applied voltage.

To ensure the cells' viability during dielectrophoresis-based experiments, tests were carried out in three cycles (Fig. 3). We captured the stretching of PBMC cells using a CCD camera, plotted the time history of cell elongation. 3 MHz frequency is chosen to have the maximum elongation in which cells are more vulnerable. First, an 8 V voltage is applied between electrodes; after 40 s, the elongation reaches its maximum stretching (Fig. 3a). Then the applied voltage difference is reduced to zero and allows the cells to relax. As can be seen from Fig. 3a, approximately half of the cells return to their original shape. We repeated the cycle four times, yet almost no significant change was observed in the displacement rhythm. Unfortunately, the DEP buffer is not the desired medium for keeping cells alive; and thus gradually hindering the accuracy of the results. Figure 3b depicted the same test when the applied voltage increased to 12 V. We did not observe significant changes after four cycles. However, when the test was repeated with 16 V applied voltage between the electrodes, a maximum elongation drop is obtained (Fig. 3c). Furthermore, the cells were not able to return to their original shapes. In some cases, lysis of cells was also observed. We conclude that applying a voltage of 16 V between the electrodes can seriously damage the cell viability. Based on this, we limit the applied voltage to 12 V, and the experimental results are sufficiently accurate. On the other hand, at higher voltages (16 V), cells are severely dented, and experimental results are not acceptable.

It should be mentioned that cell viability does not only depend on the applied voltage. One of the significant parameters is the structure of the microfluidic chip, including the geometry of electrodes. We have used the optimum Rectangular–Rectangular configuration in these experiments, which we discussed in “The optimum electrode configuration” section. Other electrodes geometry may lead to lower cell viability. For example, due to the high electric field gradient present at the triangular electrode tips, such geometry proofs are detrimental to the cells. One of the other parameters which play an essential role in cell viability is the passivation of electrodes, which we discussed in the “Fabrication” section in methods part. Definitely, without the passivation of electrodes, the direct contact between a cell and electrodes results in the electrolysis of water molecules and, consequently, reduces cells' viabilities. Also, the medium that cells are suspended in can harshly change the viability of cells. Therefore, the viability reported here is only achieved with our optimized medium discussed in the “Sample preparation” section.

### The optimum electrode configuration

**Figure 4:**
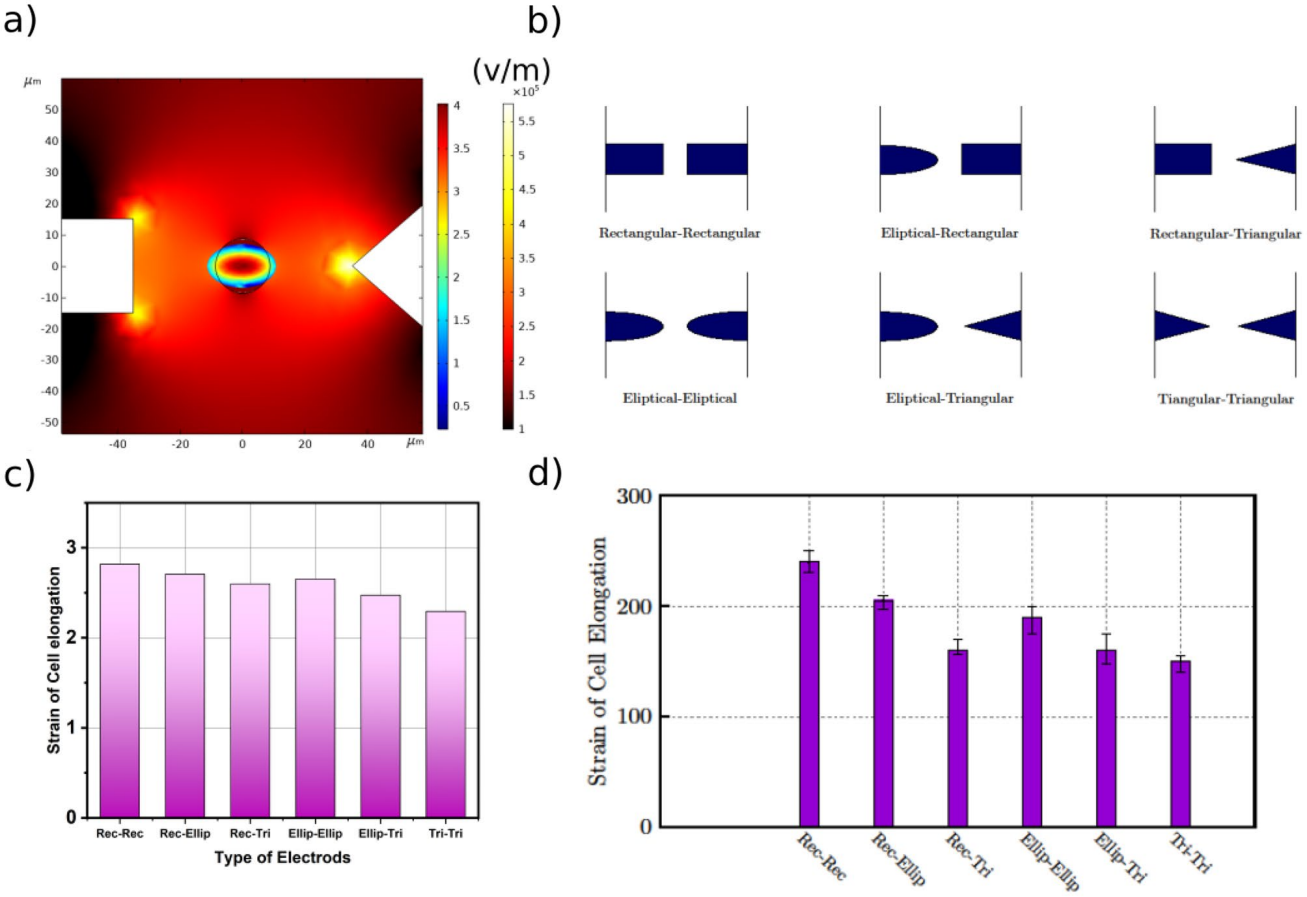
Optimum electrode configuration (**a**) An example of COMSOL simulation result for Rectangular-Triangular electrode configuration. The contour of total strain and norm of the electric field are shown in the particle and the microfluidic channel, respectively. (**b**) Different scenarios for electrode configurations. (**c**) Simulation study of maximum of cell elongation strain for different electrode configurations. (**d**) Experimental study of the strain of erythrocytes for different electrode configurations.

Various electrode configurations have already been employed in previous studies to evaluate cell mechanical properties^8,24,27,30^. An optimum electrode configuration is the one that is less detrimental to the cells and provides the largest elongation under similar conditions (applied voltage and frequency) for the same cell and medium. We studied six configurations based on different configurations studied in other literature^22–26^, including rectangular, elliptical, triangular, and combinations, depicted in Fig. 4b.

We used COMSOL Multiphysics software in conjunction with the Genetic Algorithm in MATLAB (Fig. 4a) for finding the optimum electrode configuration. In this simulation, the particle diameter is 8 um (which corresponds with the average Erythrocytes size). It should be mentioned that we redo the simulation analysis for different sizes of the particle, and the same conclusion is drawn. The Young's modulus of the particle is 0.1 kPa. Since the relation between strain and Young's modulus is not changing with simulation conditions (e.g. electrodes configuration), the conclusion about the optimum electrodes configuration is not changing with the chosen Young's modulus value. The electrodes' size is in the range of 5–50 µm, and the distance between electrodes is in the range of 20–70 µm. And the applied voltage is 10 V. The permittivity of medium and particle are 80 and 120, respectively. In the section “The fundamental equation”, we show that the effect of permittivity values on cell elongation is almost independent of electrodes' size and geometry.

First, we found an optimized dimension (sizes and distance between electrodes) for each scenario. In this case, the Genetic Algorithm in MATLAB is used. In genetic algorithm analysis, the variables are width and distance between electrodes. We considered ten generations with twenty populations for genetic algorithm analysis. The objective function (which is minimized in this study) is the reverse of cell elongation. In other words, this study aims to find the optimum width and distance between electrodes, which results in maximum cell elongation. We observed that after the 9th generation, there is less than 1 percent variation in the objective function value. We found that in all scenarios, the smaller the distance between the electrodes is, the larger the cell elongation will be. Moreover, the thicker the electrodes is, the larger the elongation will result.

Once we found the optimum width and distance between electrodes, the maximum elongation is calculated for different scenarios, using simulation and experimentally. Our simulation result shows that the rectangular-rectangular scenario results in maximum elongation (Fig. 4c). We designed and fabricated our DEP device based on our simulation results (the optimum width and distance between electrodes). The results of such a comparison are shown in Fig. 4d. Compared with other designs, rectangular electrodes provide the largest elongation, which is consistent with our simulation results. Smaller size electrodes lead to smaller chips, higher fabrication yield, as well as handling larger sample sizes. Therefore, we select rectangular–rectangular geometry as the best design.

One of the important considerations in DEP-based method for cell mechanical properties assessment is positioning of a cell in a specific area, which is required for further analysis. In the elliptical and the triangular-shaped electrodes, it is not guaranteed that the cells elongate only along the gap between electrodes; the cells can also adhere to electrodes edges and elongate perpendicularly.

### The fundamental equation

**Figure 5:**
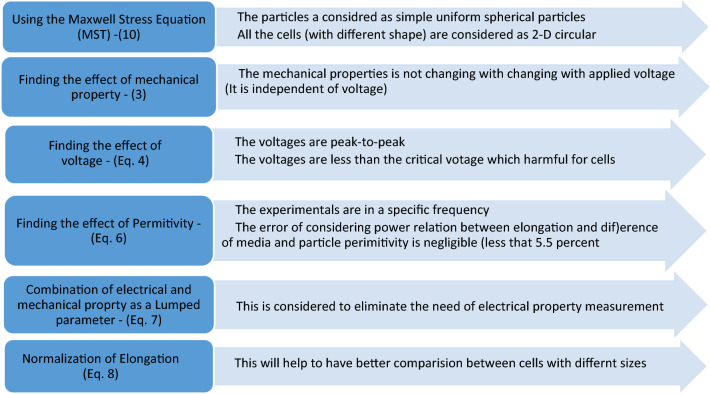
Different steps of the equation development and the assumptions in each step.

The cell elongation arising from DEP force highly depends on the electrode design. Although several methods exist for measuring cell mechanical properties, including micropipette^10^ or AFM^9^, providing a direct relationship between cell deformation and different cell parameters and the mechanical properties, limited work has been published on utilizing DEP to investigate the mechanical properties of the cell. Our research is the first study published on the subject to the best of our knowledge. The steps of developing this equation and assumptions are elaborated in Fig. 5.

We propose the following explicit equation, which segregates different effective parameters influencing cell elongation.

1 $$ f_{DEP}^{s} = \varepsilon_{m} \left( {\left( {\overline{\overline{{E_{out} }}} .\overrightarrow {n} } \right)\overline{\overline{{E_{out} }}} - \frac{1}{2}\left| {\overline{\overline{{E_{out} }}} } \right|^{2} \overrightarrow {n} } \right) - \varepsilon_{c} \left( {\left( {\overline{\overline{{E_{in} }}} .\overrightarrow {n} } \right)\overline{\overline{{E_{in} }}} - \frac{1}{2}\left| {\overline{\overline{{E_{in} }}} } \right|^{2} \overrightarrow {n} } \right) $$

where $f_{DEP}^{s}$ is the stress on the cell surface $\varepsilon_{m}$ and $\varepsilon_{c}$ represent the complex permittivity of medium and the cell, respectively. The cell's complex permittivity depends on the ratio of membrane thickness to the radius of the cell as well as permittivity and conductivity of the cell's cytoplasm and membrane^36^. Also, the effect of each of the above-mentioned parameters is inextricably intertwined with the frequency of applied electrical potential. Different complex models have been developed to measure cells' permittivity, considering the mentioned parameters for different cell types^37^.

$\overline{\overline{{E_{in} }}}$ and $\overline{\overline{{E_{out} }}}$ are the electric fields inside and outside of the cell and depend on the gap size between the electrodes, average cell radius, and the potential difference between the electrodes respectively and $\overrightarrow {n}$ is the unit vector normal to the cell surface. Hence, the stress on the cell surface depends on several parameters as shown below:

2 $$ f_{DEP}^{s} \propto \left( {\varepsilon_{m} ,\varepsilon_{c} ,R,d,w,V} \right) $$

where R is the radius of cell, w, and d are width and distance between the electrodes, respectively and V is a peak to peak potential difference between electrodes. Regarding the relation between elongation and stress $\left( {L= D \times f_{DEP}^{s} } \right)$ and the relation between strain and elongation can be rewritten as:

3 $$ L = D \times \beta \left( {\varepsilon_{m} ,\varepsilon_{c} ,R,d,w,V} \right) $$

where L is cell elongation, and D is the mechanical property. The function $\beta$ is unknown and should be calculated. We use FEA software (COMSOL 5.2 a) to find the influence of each variables of function $\beta$ on L.

**Figure 6:**
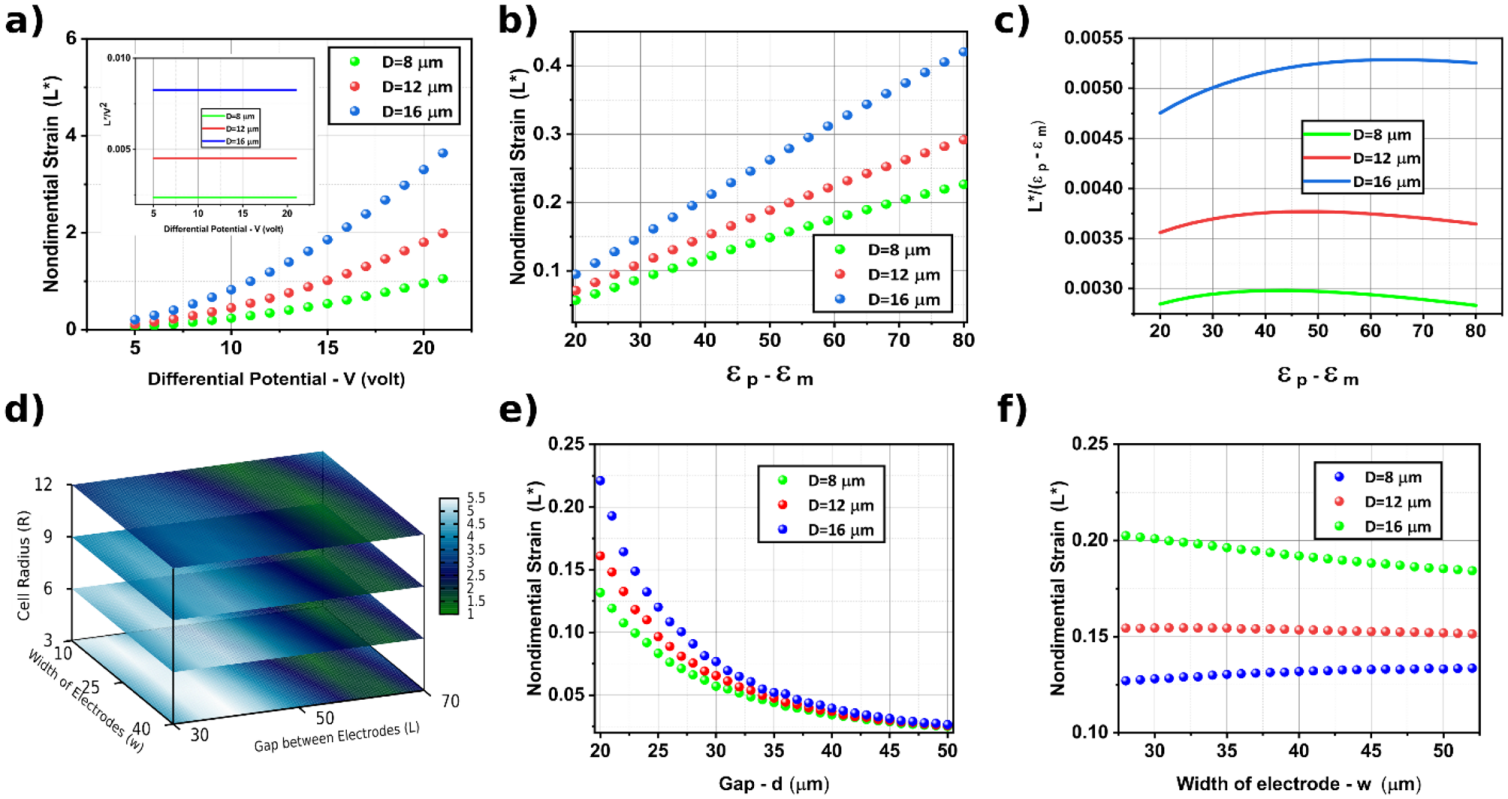
Study of the effect of the different parameters on cell elongation. (**a**) Variation of non-dimensional strain vs differential potential. The inset gives the fact that elongation is directly dependent on power two of the applied voltage. (**b**) Variation of non-dimensional strain vs the difference between medium and cell permittivity. (**c**) Normalized non-dimensional strain with the difference of medium and cell permittivity vs difference of medium and cell permittivity, which shows a nonlinear relationship. (**d**) Error expected in approximation used to simplify elongation's dependency on the permittivity of particle and medium. The error for different sizes of cells and electrodes is less than 5.5. (**e**) Variation of non-dimensional strain vs gap (**d**) Variation of non-dimensional strain vs width of electrode.

The electric field between the two electrodes is proportional to the gradient of the electrical potential $(\Phi)$. Furthermore, due to the existing inner product in, the cell elongation L is proportional to $V^{2}$ (Fig. 6a). In other words:

4 $$ L = D \times V^{2} \times G\left( {\varepsilon_{m} ,\varepsilon_{c} ,R,d,w} \right) $$

Another significant parameter that affects the cell response is the permittivity of both the medium and the cell and. The permittivity of the cells depends on its electrical properties. If the electric field inside and outside of the cell is assumed to be equal, Eq. 4 can be rewritten as:

5 $$ L = D \times V^{2} \times \left( {\varepsilon_{m} - \varepsilon_{c} } \right) \times \gamma \left( {R,d,w} \right) $$

Such an assumption is not accurate (Fig. 6b,c). If the parameter $\left( {{L \mathord{\left/ {\vphantom {L {\left( {\varepsilon_{m} - \varepsilon_{c} } \right)^{\alpha } }}} \right. \kern-\nulldelimiterspace} {\left( {\varepsilon_{m} - \varepsilon_{c} } \right)^{\alpha } }}} \right)$ is plotted against cells' size and size of electrodes, one finds that the variation of the parameter $\left( {{L \mathord{\left/ {\vphantom {L {\left( {\varepsilon_{m} - \varepsilon_{c} } \right)^{\alpha } }}} \right. \kern-\nulldelimiterspace} {\left( {\varepsilon_{m} - \varepsilon_{c} } \right)^{\alpha } }}} \right)$ is minimum for $\alpha = 0.84$ regardless of electrodes' dimensions. The maximum deviations, for various electrodes width and length and the cell radius, are depicted in Fig. 6d. The deviation is less than 5.5 percent for all configurations considered. Therefore, we can rewrite Eq. as:

6 $$ L = D \times V^{2} \times \left( {\varepsilon_{m} - \varepsilon_{c} } \right)^{0.84} \times \gamma \left( {R,d,w} \right) $$

It is thus possible to separate the electrical properties parameters from other parameters that affect the elongation. The electrical properties of cells can be replaced with previously developed models^37^. Although an explicit relation is proposed, we suggest that this effect should be merged with the parameter representing the mechanical property, D, of the cells. This can be regarded as a unique property indicative of the combination of mechanical and electrical properties. The reason is that a vast body of literature already exists which treats this issue under different specific conditions. This wide range of representing electrical properties may lead to confusion in implementing. To avoid confusion in future studies, we propose rewriting as:

7 $$ L = S \times V^{2} \times \gamma \left( {R,d,w} \right) $$

We now define the effect of the remaining three parameters R, d, w, by an experimental dimensionless function $\gamma \left( {R,d,w} \right)$ and investigate its effect on the cell elongation. The examination shows that elongation's dependency on the width and length of electrodes changes as the cell's size is varied (Fig. 6e,f). Thus, finding an explicit relation between the above parameters is not easy. We, therefore, provide a table for $\gamma$ in Table Apx-1. The results presented here are given for common cell sizes. The corresponding value of $\gamma$ other parameters R, d, and w can be obtained by interpolating the data presented in Table Apx-1.

8 $$ L^{*} = \frac{L - R}{R} = S \times V^{2} \times \gamma \left( {R,d,w} \right) $$

### Experimental verification of the proposed equation

**Figure 7:**
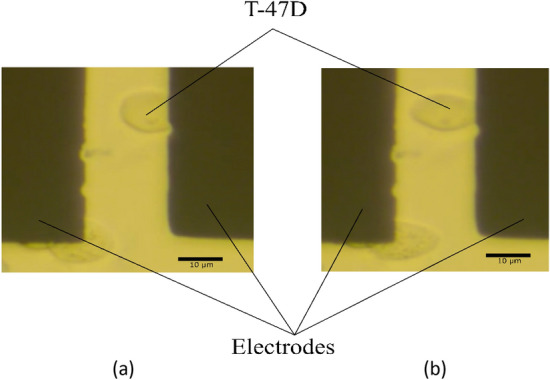
Elongation of T-47D in an electrical field is shown for different times (**a**) T = 0 s (**b**) T = 105 s.

**Figure 8:**
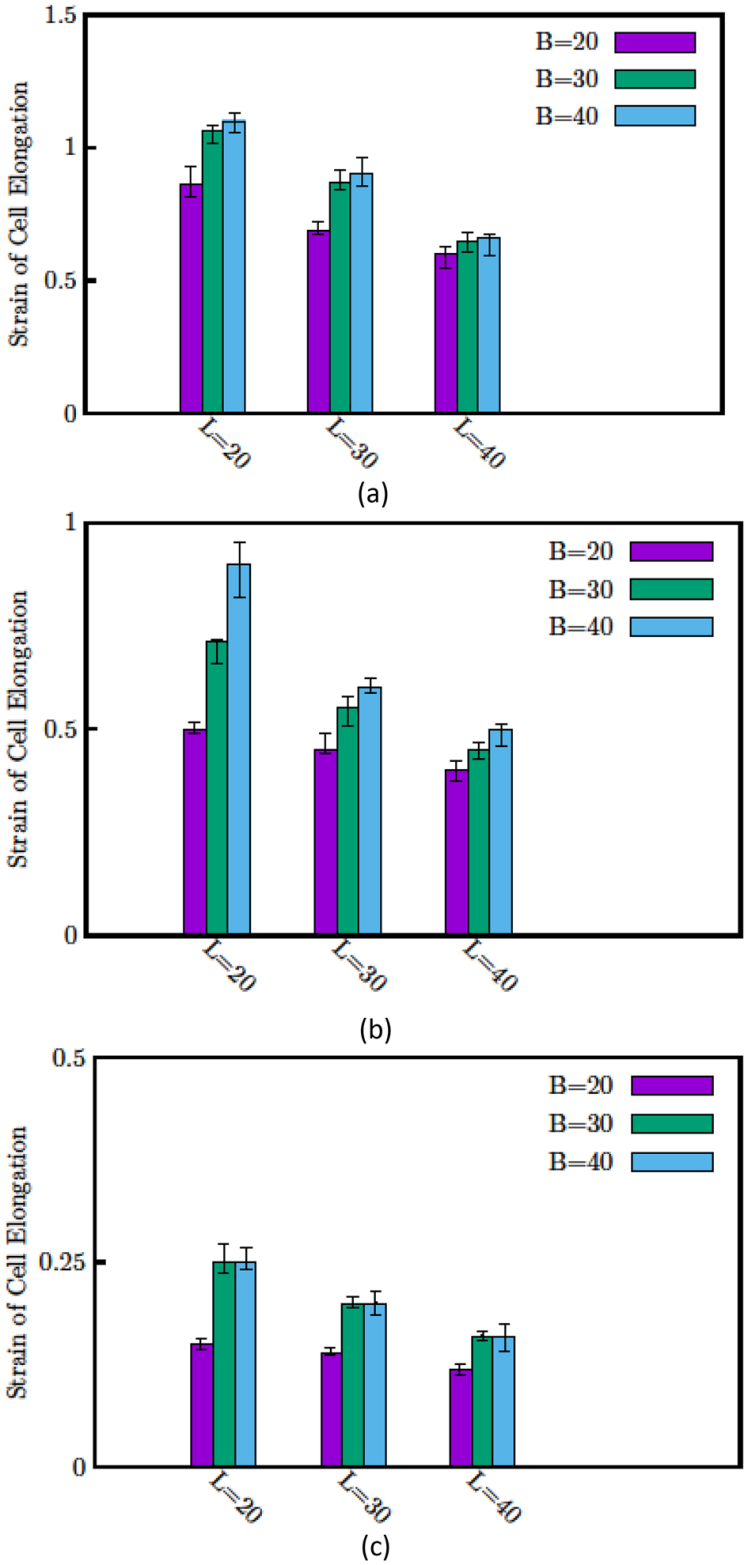
Normalize elongation ($${L}^{*})$$ of (**a**) Erythrocyte (**b**) PBMC (**c**) T-47D is shown for different sizes of electrodes. In each figure, the normalized elongation for three width and three gap sizes of electrodes (all units are micrometer).

In this section, we evaluate the accuracy of the proposed equation, under different experimental conditions. Three kinds of circulating cells' mechanical properties, which have been frequently addressed in previous work, are employed in this study. These are (1) Erythrocyte (2) PBMC (3) T-47D Breast cancer Cell line. After the preparation of cells, they are injected into a microfluidic channel. The pump is turned off as soon as all the cells are distributed inside the channel, and an A.C. voltage is applied between electrodes by employing a function generator. Due to the positive dielectrophoresis, the cells are attracted to the electrodes with a low voltage as a 1 V amplitude. However, to assure that all the cells will be attracted to the electrodes, an A.C. signal (with 2 V amplitude and 3 MHz frequency) is employed. The voltage amplitude is modified according to cell elasticity. The Erythrocytes show the fastest response while TD + 4 cells show the slowest response (The camera image of the T-47D response is shown in Fig. 7). The cells' time response is captured using a camera under an inverted microscope (Nikon model). Then, image processing is carried out using Tracker software and the time-dependent movement of the cells' tip is recorded. The results are shown in Fig. 8. For higher reliability, sixteen cells are examined for each experimental condition.

**Figure 9:**
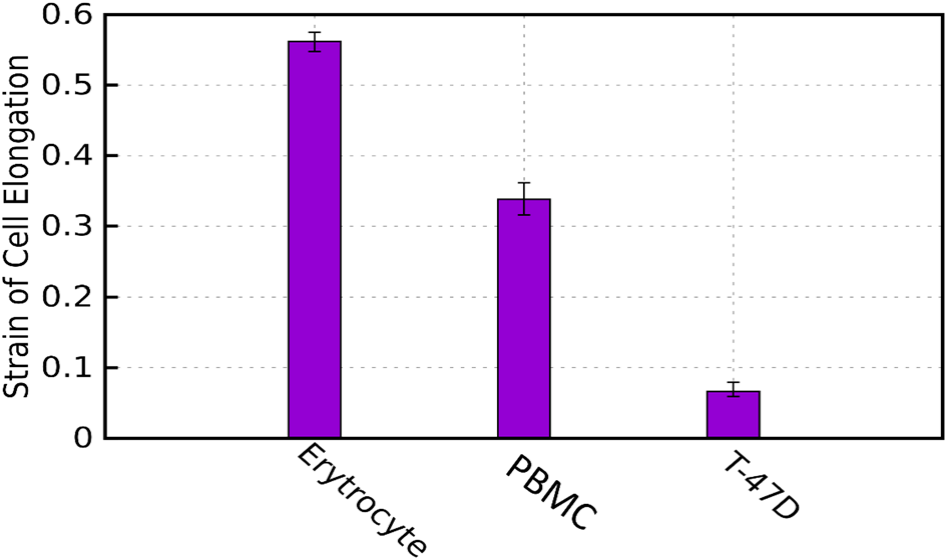
Lumped property parameter (S) for three kinds of circulating cells (Erythrocyte, PBMC, and T-47D).

Then, for each of the three cell types, $S$ is calculated. As depicted in Fig. 9, only a 5% discrepancy in S exists between the model and the experimental results, certifying the model's good accuracy. We conclude that our derived equation can precisely model different parameters and uniquely predict each cell type's Lumped properties under specific experimental conditions.

## Methods

### Model

The Dielectrophoresis phenomenon is usually modeled by one of the two main methods: A popular method is dipole effective, widely used in electrostatics literature long before introducing the term DEP. The DEP force, $f_{DEP}^{s}$ in this method, is given by:

9 $$ \left\langle {f_{DEP}^{s} } \right\rangle = 2\pi r^{3} \varepsilon_{0} \varepsilon_{m} Rr\left[ {K\left( \omega \right)^{*} } \right]\nabla \left| E \right|^{2} $$

where $\varepsilon$ is the permittivity, $\omega$ is the angular frequency of the applied electric field, and $K\left( \omega \right)^{*}$ is the Clausius–Mossotti factor:

10 $$ K\left( \omega \right)^{*} = \frac{{\varepsilon_{p}^{*} - \varepsilon_{m}^{*} }}{{\varepsilon_{p}^{*} + 2\varepsilon_{m}^{*} }} $$

where $\varepsilon_{i}^{*} = \varepsilon_{i} - j\frac{\sigma }{\omega }$ is the complex permittivity and $\sigma$ is the conductivity.

In this method, the particle is considered as a single dipole. Although the method has shown acceptable results in many cases, it is not suitable for cases where a cell deforms due to the following reasons:

1. The particle deformation is not taken into account. The variation of forces along the particle's boundary is neglected.
2. Due to the small gap between the electrodes compared to the cell's diameter, the electrodes' electric field is highly disturbed upon the cells' presence.
3. Although cells experience an elongation along the uniform electric field, the DEP force given by Eq. 9, predicts no DEP force due to the uniform electric field's assumption.

Therefore as far as soft biomaterials are concerned, a more general formulation is needed to model this phenomenon to consider the effect of the said parameters that are neglected in Eq. 9. The most general equation for considering the electrical forces is the Maxwell Stress Tensor (MST). In the absence of a magnetic field, the element of which is given as:

10 $$ \sigma_{ij} = \varepsilon \left( {E_{i} E_{j} - \frac{1}{2}\left( {\delta_{ij} E^{2} } \right)} \right) $$

where $\sigma$ is mechanical stress, and $\delta$ is the Kronecker delta. We have used Finite Element Analysis (FEA) in the multiphysics software (COMSOL version 5.2 a). In this modeling, both Electrostatic and Structural mechanics modules are employed. All the simulations are done on the 2-D surface of electrodes. First, in Electrostatics modules, MST is solved, and the boundary forces are calculated, then the calculated forces are used in the structural mechanic's module. Validation studies were successfully carried out. For the validation of results, we first performed a mesh independence study. Then, we compared our FEA results with analytical results for a simple case of a sphere in a uniform electric field.

As mentioned earlier, a dielectrophoresis-based microfluidic chip is used to validate the above mentioned numerical model. The chip consists of two parts: A substrate with Ti/Au electrodes patterns and a microfluidic channel fabricated. In this study, we used the cells detected in a blood sample, i.e. red blood cells and Peripheral blood mononuclear cells (PBMC) and a breast cancer cell line (T-47D). The authors confirm ethical approval that all the experimental methods for biological tests are carried out following relevant guidelines, and the University approves them of Tehran. Also, informed consent was obtained from all participants.

### Fabrication

**Figure 10:**
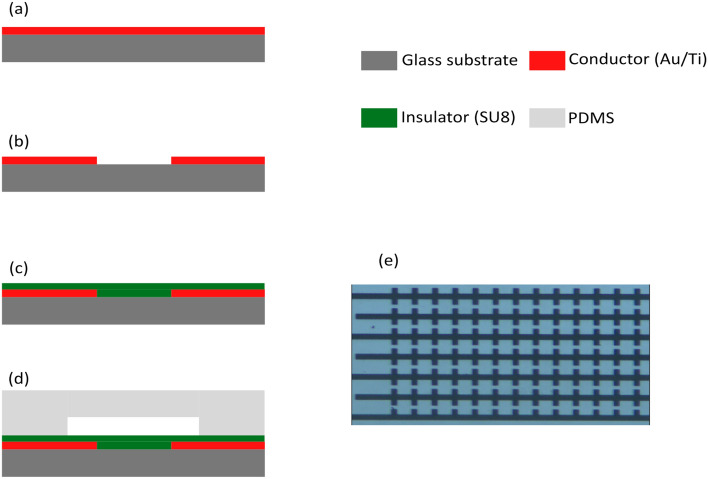
Fabrication flow (**a**) A conductive layer (Au/Ti) coating on a glass substrate, (**b**) Patterning of electrodes using Photo-lithography mask, (**c**) Coating of insulator layer (SU8), (**d**) Fabrication of microfluidic channel by soft lithography (**e**) Microscopic image of fabricated electrodes on the glass substrate.

The substrate is first cleaned using the RCA method. A Titanium layer is then evaporated on the glass surface with a Physical Vapor Deposition (PVD) system (Y.N.Saleh CO.) to facilitate the subsequent Au's adhesion to the substrate. Then, a layer of gold is deposited on the surface of Titanium covered glass using a D.C. sputtering system (Fig. 10a). The electrodes are then patterned on the surface using the standard photolithographic technique (Fig. 10b). At low frequencies (lower than 100 kHz) direct contact between electrodes and the fluid leads to bubbles. In previous studies^25,26^, hydrogenated silicon nitride has been used as a passivation layer to avoid the formation of bubbles. Here, we used diluted SU8 (MicroChem) by cyclopentanone as a passivation layer (Fig. 10c). The SU8 is a well-known biocompatible material and is more reliable than the traditional method proposed in^26^. Yet coating a SU8 layer on the surface is much faster than the deposition of hydrogenated silicon nitride. Furthermore, an extra photolithographic step is needed to remove the excess hydrogenated silicon nitride deposited layer; therefore, the proposed process is much simpler since SU8 is a negative photoresist. The experimental results show that the system can be used for at least 5 min without any bubble formation. Next, a (Polydimethylsiloxane) PDMS microfluidic channel is prepared using the standard soft lithographic technique on a separate glass substrate (Fig. 10d). The two parts are then bonded together using an oxygen plasma system, and electrical contacts are made. Finally, microfluidics inlet and outlet are connected. Figure 10e illustrates the chip layout.

### Sample preparation

The PBMC cells were isolated from a whole blood sample using the Ficoll method. There are three range sizes of cells in a sample: Erythrocytes (6 < R < 8), most PBMC (9 < R < 12), and CTCs (R > 12). After diluting the sample, the cells were washed with phosphate-buffered saline (PBS). Then, the cells were suspended again in a low conductive medium. The selection of a proper medium is a crucial issue in the DEP method. An appropriate medium should have a low conductivity to decrease the effect of Joule heating and increase the possibility of working in low frequencies. Furthermore, it should provide proper osmotic pressure for cells' viability, low viscosity to decrease the damping effect on the movement of cells, and bare toxic properties for the cells. In previous studies, a medium has been proposed and has shown acceptable results. The medium constituents are presented in Table 1. The existence of glucose slightly increases the viscosity of the medium. However, after the fluid flow stops, the glucose rapidly deposits on the substrate's surface. Cells may then penetrate this glucose layer. The free movement of cells is thus obstructed. This problem may be reduced to some extent by adding a small amount of Polysorbate 80. Finally, to obtain reliable results, the medium should be freshly prepared just before carrying the actual experiment.

**Table 1:** DEP buffer constituents.

Sucrose	$$8.5\left( {{\raise0.5ex\hbox{$\scriptstyle g$} \kern-0.1em/\kern-0.15em \lower0.25ex\hbox{$\scriptstyle L$}}} \right)$$
Dextrose	$$0.3\left( {{\raise0.5ex\hbox{$\scriptstyle g$} \kern-0.1em/\kern-0.15em \lower0.25ex\hbox{$\scriptstyle L$}}} \right)$$
CaCl	$$20\left( {{\raise0.5ex\hbox{$\scriptstyle {mg}$} \kern-0.1em/\kern-0.15em \lower0.25ex\hbox{$\scriptstyle L$}}} \right)$$
Polysorbate 80	$$1\left( {{\raise0.5ex\hbox{$\scriptstyle {mL}$} \kern-0.1em/\kern-0.15em \lower0.25ex\hbox{$\scriptstyle L$}}} \right)$$

The selection of the DEP buffer is a delicate issue. Some of the most noteworthy issues which restrict the selection of DEP buffer is:

- The sensitivity of cells to their environments such as P.H., Osmotic pressure, toxicity, and viscosity.
- Providing efficient DEP force.
- Joule heating due to the conductivity of the solution.

Therefore, there is only a narrow range of choices. In previous DEP studies, a medium consisting of a mixture of deionized (DI) water and sucrose/dextrose with a relative permittivity of 80 has widely been used^29^.

## Conclusions

We have shown that among several electrode designs previously reported, rectangular-rectangular serves as an optimum configuration for obtaining the highest cell elongation assuming other conditions remain constant. Furthermore, we have modeled the cell elongation using a novel analytical method and verified our model with our experimental results. The model was examined for three kinds of circulating cells; namely, Erythrocyte, PBMC, and breast cancer cell line (T-47D). We successfully showed that the fundamental equation can provide a quantitative parameter indicative of the intrinsic properties of the cells. This parameter can be employed in a high throughput system to distinguish between different types of cells or be considered as a lumped parameter showing cells' electrical and mechanical properties. Furthermore, it alleviates difficulties often seen in other methods including cell trapping, avoids complicated test procedures, and low accuracy, while enjoys the on-chip realization and provides label-free analysis for a variety of cells with different sizes and properties.

## Supplementary Information

Supplementary Information
