# Supplementary material for: Cell properties assessment using optimized dielectrophoresis-based cell stretching and lumped mechanical modeling
Source: Sci Rep. 2021 Jan 27;11:2341. doi: 10.1038/s41598-020-78411-1 (PMC7840762; doi:10.1038/s41598-020-78411-1)
Supplement: Supplementary file 1 — Supplementary Information [file 41598_2020_78411_MOESM1_ESM.docx]

**Characterization of cell mechanical properties using optimized dielectrophoresis-based cell stretching and lumped modeling**

Imman Isaac Hosseini ^+^, Mahdi Moghimi Zand ^+*^, Amir Ali Ebadi^‡^, Morteza Fathipour^‡^

^+^ Small Medical Devices, BioMEMS & LoC Lab, Department of Mechanical Engineering, College of Engineering, University of Tehran, Postal Code 14399-55961, Tehran, Iran

^‡^ MEMS & NEMS Lab, School of Electrical and Computer Engineering, Faculty of Engineering, University of Tehran, Tehran, Iran

^*^Corresponding Author: [mahdimoghimi@ut.ac.ir](mailto:mahdimoghimi@ut.ac.ir)

**Appendix**

Table Apx-1. __parameter for different Thickness, the width of electrode and size of particles

| D=8 | | | | | |
| --- | --- | --- | --- | --- | --- |
|  | B=10 | B=20 | B=30 | B=40 | B=50 |
| d=15 | 0.1383 | 0.1542 | 0.1621 | 0.171 | 0.1714 |
| d=20 | 0.1249 | 0.1489 | 0.1554 | 0.1599 | 0.1602 |
| d=25 | 0.1154 | 0.1322 | 0.1398 | 0.1424 | 0.1428 |
| d=30 | 0.1012 | 0.1256 | 0.1278 | 0.1318 | 0.132 |
| d=40 | 0.0857 | 0.0941 | 0.0984 | 0.1011 | 0.1011 |
| D=12 | | | | | |
|  | B=10 | B=20 | B=30 | B=40 | B=50 |
| d=15 | 0.0813 | 0.1131 | 0.1284 | 0.1327 | 0.1333 |
| d=20 | 0.0782 | 0.1022 | 0.1135 | 0.1189 | 0.1212 |
| d=25 | 0.0742 | 0.0971 | 0.1001 | 0.1109 | 0.112 |
| d=30 | 0.0679 | 0.0814 | 0.0892 | 0.0921 | 0.0925 |
| d=40 | 0.0593 | 0.0686 | 0.0721 | 0.0751 | 0.0754 |
| D=16 | | | | | |
|  | B=10 | B=20 | B=30 | B=40 | B=50 |
| d=15 | - | - | - | - | - |
| d=20 | 0.0583 | 0.0833 | 0.0912 | 0.0998 | 0.1065 |
| d=25 | 0.0542 | 0.0801 | 0.884 | 0.964 | 0.0982 |
| d=30 | 0.0489 | 0.0683 | 0.0722 | 0.0809 | 0.812 |
| d=40 | 0.0426 | 0.0564 | 0.0598 | 0.06 | 0.0604 |
| D=20 | | | | | |
|  | B=10 | B=20 | B=30 | B=40 | B=50 |
| d=15 | - | - | - | - | - |
| d=20 | - | - | - | - | - |
| d=25 | 0.0545 | 0.0801 | 0.09015 | 0.0994 | 0.1005 |
| d=30 | 0.482 | 0.0672 | 0.0705 | 0.0748 | 0.0752 |
| d=40 | 0.0378 | 0.0478 | 0.0545 | 0.056 | 0.0565 |
